# Robust CXCL10/IP-10 and CCL5/RANTES Production Induced by Tick-Borne Encephalitis Virus in Human Brain Pericytes Despite Weak Infection

**DOI:** 10.3390/ijms25147892

**Published:** 2024-07-18

**Authors:** Veronika Prančlová, Václav Hönig, Marta Zemanová, Daniel Růžek, Martin Palus

**Affiliations:** 1 Institute of Parasitology, Biology Centre of the Czech Academy of Sciences, CZ-37005 Ceske Budejovice, Czech Republichonig@paru.cas.cz (V.H.);; 2 Faculty of Science, University of South Bohemia, CZ-37005 Ceske Budejovice, Czech Republic; 3 Laboratory of Emerging Viral Infections, Veterinary Research Institute, CZ-62100 Brno, Czech Republic; 4 Department of Experimental Biology, Faculty of Science, Masaryk University, CZ-62500 Brno, Czech Republic

**Keywords:** tick-borne encephalitis virus, human pericytes, infection, chemokine, flavivirus, CXCL10, CCL5, inflammation

## Abstract

Tick-borne encephalitis virus (TBEV) targets the central nervous system (CNS), leading to potentially severe neurological complications. The neurovascular unit plays a fundamental role in the CNS and in the neuroinvasion of TBEV. However, the role of human brain pericytes, a key component of the neurovascular unit, during TBEV infection has not yet been elucidated. In this study, TBEV infection of the primary human brain perivascular pericytes was investigated with highly virulent Hypr strain and mildly virulent Neudoerfl strain. We used Luminex assay to measure cytokines/chemokines and growth factors. Both viral strains showed comparable replication kinetics, peaking at 3 days post infection (dpi). Intracellular viral RNA copies peaked at 6 dpi for Hypr and 3 dpi for Neudoerfl cultures. According to immunofluorescence staining, only small proportion of pericytes were infected (3% for Hypr and 2% for Neudoerfl), and no cytopathic effect was observed in the infected cells. In cell culture supernatants, IL-6 production was detected at 3 dpi, together with slight increases in IL-15 and IL-4, but IP-10, RANTES and MCP-1 were the main chemokines released after TBEV infection. These chemokines play key roles in both immune defense and immunopathology during TBE. This study suggests that pericytes are an important source of these signaling molecules during TBEV infection in the brain.

## 1. Introduction

Tick-borne encephalitis virus (TBEV), *Orthoflavivirus encephalitidis*, is a human neurotropic pathogen transmitted mainly through the bite of an infected tick. While less than 2% of tick-borne encephalitis (TBE) cases in Europe are fatal, long-term sequelae occur in about 40–50% of patients who have developed a neurological phase of TBE [1,2].

In order to develop a neurological phase of TBE, the virus must cross the blood–brain barrier (BBB). The BBB is a highly selective, semi-permeable barrier that separates the circulating blood from the brain and the extracellular fluid in the central nervous system (CNS). It is composed of neurovascular unit (NVU) cells: tightly interconnected endothelial cells lining the capillaries of the brain, astrocyte end feet and brain pericytes. The microvascular endothelial cells are interconnected by tight junctions (TJ), which are responsible for the paracellular barrier in the capillaries perfusing the brain. TJ proteins include claudins and occludin, which are linked to the cytoskeleton by cytoplasmic proteins such as zonula occludens (ZOs) [3]. Astrocytes have their end feet in close contact with the basal membrane, while their other projections extend further into the brain parenchyma and interact with other cells and components of the nervous tissue [4]. Pericytes support and maintain the endothelium through both paracrine signaling and contact-mediated interactions [5]. Their importance in maintaining the integrity of the BBB has been demonstrated using adult mice with pericyte deficiency, in which reduced pericyte coverage increased BBB permeability and the accumulation of water, plasma proteins and various tracers in the brain [6].

Previous studies of TBEV infection within the human neurovascular unit have examined various cell types, including human brain microvascular endothelial cells (HBMECs) [7], astrocytes [8,9,10], microglial cells [11] and neurons [12], all of which support productive viral infection. In vitro experiments using a monoculture model of the human BBB suggest that HBMECs may facilitate TBEV’s entry into the brain without compromising the integrity of the blood–brain barrier BBB [7]. Infected astrocytes activate and release proinflammatory cytokines that may contribute to neurotoxicity and BBB breakdown during TBE [8,13]. TBEV infection of human neurons increases viral replication after the stimulation of autophagy, etc. [12]. Although pericytes form a neurovascular complex with HBMECs and are the second cell type with which the virus comes into contact on its way from the blood to the brain parenchyma, their role in TBE pathogenesis has been largely overlooked.

Pericytes are susceptible to infection with some mosquito-borne viruses, namely dengue virus (DENV), Japanese encephalitis virus (JEV) and Zika virus (ZIKV), which belong to the same genus as TBEV [14]. In the case of JEV, the infection of the pericytes can trigger the production of inflammatory molecules such as IL-6, RANTES (also known as CCL5) and PGE2. Furthermore, the integrity of the endothelium has been shown to be negatively affected not only when treated with supernatants from JEV-infected pericytes but also when established co-cultures of endothelial cells and pericytes are inoculated with JEV [15]. Similarly, ZIKV infection of human pericytes has been associated with the compromised integrity of the epithelial layers of the human choroid plexus [16].

This study aims to investigate the particular contribution of human brain vascular pericytes (HBVPs) to both TBEV infection and subsequent neuropathological processes.

## 2. Results

### 2.1. Human Pericytes Are Susceptible to TBEV Infection

**Figure 1:**
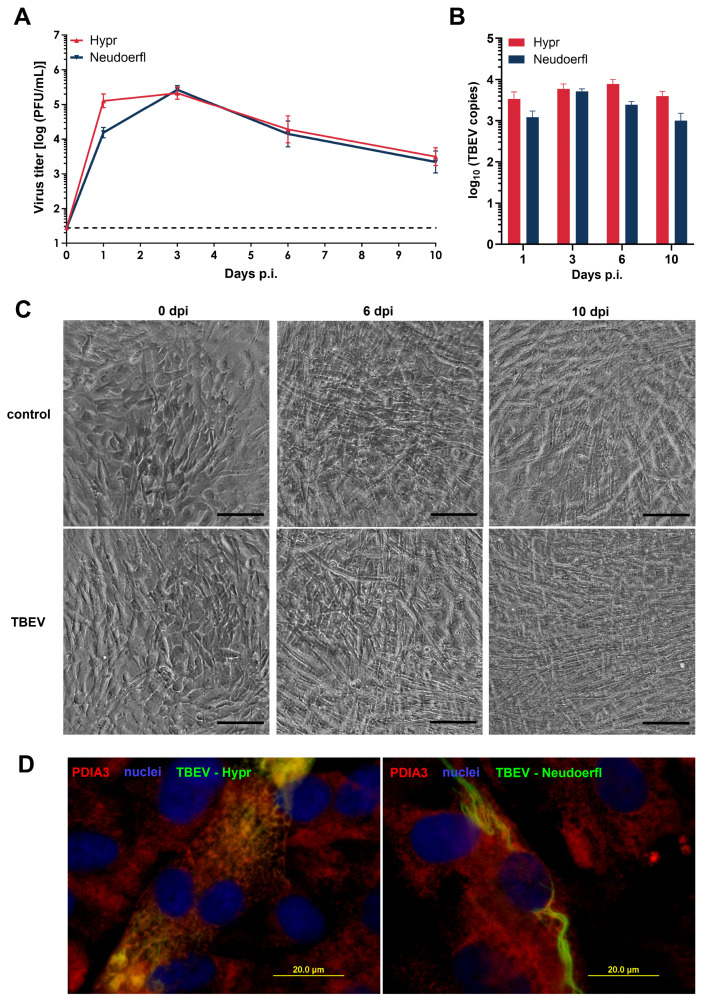
TBEV-infected HBVP. (**A**) Virus titers in cell-free supernatants of infected HBVP were evaluated on days 0, 1, 3, 6 and 10 p.i. by plaque assay. The results represent the mean ± SEM of 3 independent experiments that were run in biological triplicates. (**B**) The number of intracellular TBEV RNA copies was evaluated on days 1, 3, 6 and 10 p.i. by qRT-PCR. The results represent the mean ± SEM of 2 independent experiments that were run in biological triplicates. (**C**) HBVP were infected with TBEV strain Hypr and examined for signs of CPE using light microscopy (scale: 100 µm). (**D**) HBVP grown on slides were infected with the TBEV strains Hypr and Neudoerfl. At 3 days p.i., cells were fixed and stained with anti-PDIA3 (red) and anti-flavivirus envelope protein antibody (green). Nuclei counterstained with DAPI are shown in blue (scale: 20 µm).

To characterize TBEV infection of human pericytes, we infected human brain perivascular pericytes (HBVPs) with two viral strains, highly virulent Hypr and mildly virulent Neudoerfl. These two strains were used because they are the most studied and characterized European TBEV strains, frequently used in similar studies as representative models of highly and mildly virulent TBEV cases [17]. Cell-free supernatants harvested at different time points post infection were investigated to determine virus titers by plaque assay. Replication kinetics were shown to be strikingly similar for both strains. In both cases, the production of new infectious viral particles was detected already at 1 day p.i., with TBEV strain Hypr showing a slightly sharper increase in virus titer compared to the strain Neudoerfl. The peak of viral production was observed 3 days p.i., when TBEV Hypr reached a maximum titer 5.33 ± 0.17 log10 PFU/mL and TBEV Neudoerfl reached 5.42 ± 0.12 log10 PFU/mL. The peak was then followed by a slight decrease in virus titer; however, it did not fall below 3.34 log10 PFU/mL (Figure 1A).

Viral replication was also quantified by RT-qPCR using cell lysates of TBEV-infected cells. Overall, the dynamics of both strains were comparable, although a slightly higher number of intracellular TBEV RNA copies was observed in the case of the more virulent strain Hypr. In the case of the strain Hypr, the number of intracellular viral RNA copies reached its peak at 6 days p.i. with 5169.2 ± 750.854, while the TBEV strain Neudoerfl reached its peak at 3 days p.i. with 7762.5 ± 2271.2 viral RNA copies per well (Figure 1B).

When observing TBEV-infected pericytes using phase-contrast microscopy, there were no signs of cytopathic effect (CPE). Even in the case of highly virulent strain Hypr, no morphological changes and no cell death were noticeable throughout the whole course of the infection (Figure 1C).

**Figure 2:**
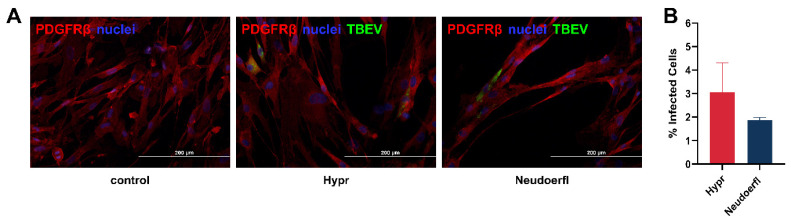
TBEV-infected human brain vascular pericytes (HBVPs). (**A**) HBVP grown on slides were infected with 2 different strains of TBEV, Hypr and Neudoerfl. At 3 days post infection (p.i.), cells were fixed and stained with anti-PDGFRβ (red) together with anti-flavivirus envelope protein antibody (green) and counterstained with DAPI (blue). (**B**) The percentage of infected cells at 3 days p.i. was determined after immunofluorescence staining. The results represent mean ± SEM based on a total of at least 1500 cells counted per viral strain.

Immunofluorescence staining was performed to survey the distribution of TBEV antigen by staining TBEV E protein together with protein disulphide isomerase family A, member 3 (PDIA3), suggesting that the viral antigen was localized in the hypertrophied and rearranged endoplasmic reticula of the cells as early as 3 days p.i. (Figure 1D). To quantify the portion of infected cells in the population, TBEV-infected HBVPs were also stained for TBEV antigen together with PDGFRβ, one of the pericyte markers. Based on a total number of at least 1500 cells counted for each viral strain, approximately 3% of HBVP cells positive for PDGFRβ were infected with the strain Hypr and approximately 2% of the cells were infected with the strain Neudoerfl at 3 days p.i. (Figure 2).

### 2.2. TBEV Infection Induces Increased Production of Oas1, IP-10/CXCL10, IFNB and IL-1B at mRNA Level

**Figure 3:**
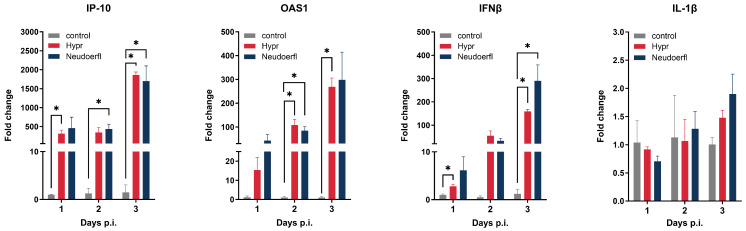
TBEV-induced changes in the expression of selected genes on an mRNA level. Total RNA isolated from TBEV-infected and uninfected control HBVP harvested at 1, 2 and 3 days p.i. was used to determine the fold changes in IP-10, OAS1, IFNβ and IL-1β with RT-qPCR. Changes in the mRNA levels were first normalized to the expression of house-keeping genes (human ACTB and GAPDH). The fold change in the infected cells was calculated compared to the corresponding controls. Data are expressed as means ± SEM. Statistically significant changes are marked with asterisk (*, *p* < 0.05).

Since viral infections induce changes in the expression of many genes, especially genes encoding various inflammatory molecules, we investigated the effect of TBEV infection on the mRNA expression of a few selected factors involved in the activation and regulation of inflammatory and immune responses, namely IP-10 (also known as CXCL10), IFNb, IL-1 and OAS1. With the sole exception of IL-1β, we observed a time-dependent increase in mRNA production for all the surveyed targets. The most dramatic upregulation was seen in the chemokine IP-10 mRNA. The upregulation was detected already at 1 day p.i. in Neudoerfl-infected HBVP and reached an almost 2000-fold change at 3 days p.i. in both Neudoerfl- and Hypr-infected cells (Figure 3).

### 2.3. Chemokines IP-10 and RANTES Are the Most Elevated Chemokines during TBEV Infection

**Figure 4:**
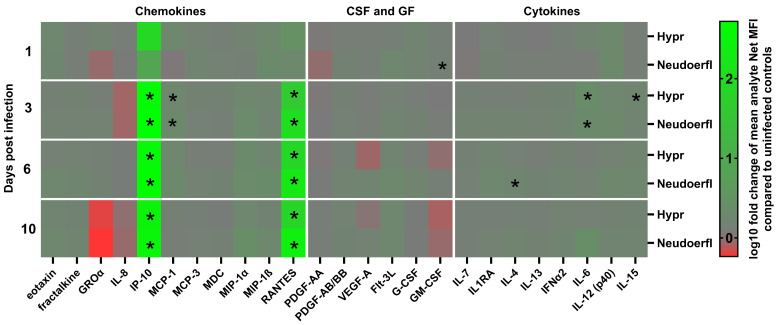
TBEV infection induces changes in cytokine/chemokine production. Cell-free supernatants of HBVP infected with the TBEV strains Hypr and Neudoerfl were harvested at 1, 3, 6 and 10 days p.i. and used for the detection of different cytokines, chemokines and growth factors by a micro-bead-based multiplex assay. The results represent the mean of the biological triplicates. Statistically significant changes compared to the control were identified using multiple unpaired *t*-tests with Benjamini, Krieger and Yekutieli two-stage step-up false-discovery-rate correction (Q = 2%). Statistically significant differences are marked by asterisks.

**Figure 5:**
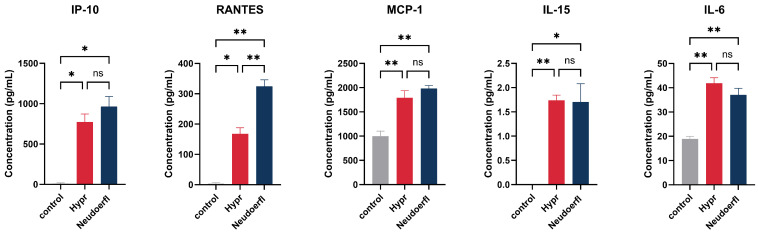
TBEV-induced production of selected chemokines/cytokines. Cell-free supernatants of TBEV-infected and uninfected control HBVP were harvested at 3 days p.i. and used for the detection of selected chemokines and cytokines by a micro-bead-based multiplex assay in order to compare the degree of their secretion at the peak of viral production. The results represent the mean of biological triplicates. Statistically significant changes compared to non-infected controls were identified using the Brown–Forsythe and Welch ANOVA tests. Statistically significant differences are marked by asterisks (* *p* < 0.05; ** *p* < 0.01; ns—not significant).

To investigate the immune response of human pericytes to TBEV infection on protein level, we analyzed cell-free supernatants of TBEV-infected HBVP using a micro-bead-based multiplex assay that allowed us to test an array of 41 different cytokines, chemokines and other modulatory factors. Samples were collected at 1, 3, 6 and 10 days p.i. to account for both early and late infection time points (Figure 4). Of the 36 analytes tested, a considerable amount was shown to be under the limit of detection and was thus excluded from the statistical analysis. The analytes that HBVP did not produce were TGFα, sCD40L, IL-1α, IL-1β, IL-2, IL-3, IL-5, IL-9, IL-10, IL-12p70, IL-17A, IFNγ, TNFα and TNFβ. In addition, EGF and FGF-2 were excluded from the statistical analysis due to their default presence in culture medium. TBEV-induced production of selected chemokines/cytokines (IP-10, RANTES, MCP-1, IL-15, IL-6) is shown in Figure 5; the rest of the analytes are presented in Supplementary Figures S1–S3.

Of all the analytes tested, the chemokines IP-10 and RANTES stood out the most with their significant increase in production in TBEV-infected pericytes compared to uninfected controls. Regardless of the virus strain, their expression increased at 3 days p.i. and remained elevated until day 10 p.i. (Figure 4). While no significant differences were observed in the production of IP-10 between the virus strains, RANTES production was significantly higher in cells infected with the strain Neudoerfl than in cells infected with the more virulent strain Hypr (Figure 5). In addition, significantly increased production of another chemokine, MCP-1, was observed at 3 days p.i. (Figure 4). Regarding the amounts of secreted chemokines, MCP-1 reached the highest concentrations of nearly 2000 pg/mL, followed by IP-10 and RANTES. (Figure 5).

Of the remaining analytes, including cytokines, colony-stimulating factors and growth factors, we detected significantly increased production of three cytokines. At 3 days p.i., TBEV-infected pericytes increased IL-6 production compared to the uninfected control (Figure 4). In the case of the strain Hypr, slightly increased production of IL-15 was also detected at this time point. Additionally, enhanced expression of IL-4 was detected at 6 days p.i. by cells infected with the strain Neudoerfl.

## 3. Discussion

**Figure 6:**
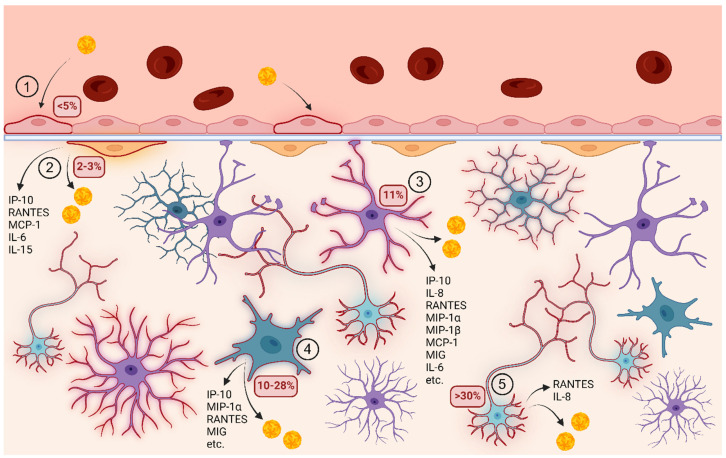
The suggested entry and spread of TBEV in the human brain and TBEV-induced cytokine/chemokine production at the protein level by the cells of the neurovascular unit on protein level. (**1**) The infectious TBEV particles present in the bloodstream infect brain microvascular endothelial cells (less than 5%) without affecting BBB integrity [7]. (**2**) TBEV further infects a small percentage of pericytes (2–3%) in which the virus replicates effectively and induces the secretion of the chemokines IP-10, RANTES, MCP-1, IL-6 and IL-15. (**3**) TBEV infection of astrocytes is associated with a higher number of infected cells (about 11%), as well as their activation and increased production of cytokines/chemokines, namely IP-10, IL-8, RANTES, MIP-1a, MIP-1B, MCP-1, MIG and IL-6 [8,13]. (**4**) Microglia are also sensitive to TBEV infection with 10–28% of the cells being infected. The infection induces the upregulation of the chemokines IP-10, MIP-1a, RANTES and MIG; however, other inflammatory molecules are produced depending on the specific viral strains. CNS-resident macrophages capable of phagocytosis, microglia are actively involved in combating the infection [11]. (**5**) Neurons, the primary target of TBEV, are highly susceptible to TBEV infection, which causes their irreversible damage and cell death. The virus is able to infect between 30 and 100% of neurons present in population [9,12,20]. Neuronal response to TBEV infection and related injury might involve the production of RANTES and IL-8 [13]. It should be noted that the percentages of infectivity for the individual cell types are based on in vitro experiments and may not accurately reflect the actual conditions in the brain.

Pericytes are an essential component of the BBB, which lies between the vascular system and the brain parenchyma (Figure 6). Their high density and close association with endothelial cells and astrocytes are essential for the maintenance of selective tight junctions and the integrity of the barrier [18]. The loss of pericytes in the brain is associated with the occurrence of neurodegenerative and neuroinflammatory diseases [19]. The role of pericytes in health and disease, including viral infections, has gained importance. Viral infections of pericytes, particularly those associated with the BBB, are common and suggest that these cells play an important role in the neurological effects induced by various viruses [14]. These infections often lead to changes in inflammatory responses, emphasizing the importance of pericytes for understanding the general impact of immune system dysfunction on viral disease.

Interactions between pericytes and viruses have been described for several human pathogens, including SARS-CoV-2 [21], human cytomegalovirus (HCMV) [22], the human immunodeficiency virus (HIV-1) [23], mosquito-borne orthoflaviviruses such as DENV [24], JEV [15], ZIKV [16] and the tick-borne Powassan virus (POWV) [25]. However, the interactions between pericytes and TBEV are not yet fully understood. The aim of this study is to describe the infection of primary human pericytes with TBEV in vitro, focusing on the kinetics of the virus, the susceptibility of the cells to infection and their immune response.

Our study shows that HBVPs are susceptible to TBEV infection, as demonstrated by successful infection with the highly virulent Hypr and the mildly virulent Neudoerfl strain. Both strains exhibited similar replication kinetics within the pericytes, with virus replication being detected as early as one day p.i. and peaking after three days p.i., indicating efficient virus replication within these cells. Even after ten days p.i., the TBEV infection did not lead to CPE or morphological changes in the cell culture recognizable by light microscopy. Despite the low proportion of infected cells (3% in Hypr and 2% in Neudoerfl), TBEV reached high viral titers of 10^5^ and 10^6^ PFU/mL, respectively. These results are comparable to TBEV-infected primary HBMEC [7], in which the viral titers also reached a similar titer and less than 5% of the cells were infected.

Pericytes may facilitate viral spread in the CNS by providing a long-term source of progeny virus, as shown by viral titers and the presence of viral RNA at late infection intervals. The role of pericytes in invading the brain parenchyma has also been hypothesized for other orthoflaviviruses such as ZIKV. In vitro, ZIKV infection of HBVPs causes the increased permeability of the BBB endothelial barrier despite a low number of ZIKV-positive cells (4%), suggesting that infection of pericytes may facilitate CNS invasion [16]. In mouse models, ZIKV infects pericytes in the choroid plexus and meninges before spreading to the cortex [16].

Pericytes respond to viral pathogens by secreting various cytokines, chemokines and growth factors, with responses varying by pathogen and pericyte subtype [26]. Our study showed a time-dependent increase in the mRNA expression of antiviral defense genes (IP-10, IL-1β, IFN-β and OAS1) in HBVPs after TBEV infection, suggesting the progressive activation of antiviral pathways. These transcriptional changes are similar to those observed in POWV-infected HBVPs [25]. Despite increased IFN-β expression, TBEV-infected HBVPs established persistent infection, in contrast to DENV, which is eliminated from infected human endothelial cells by IFN-β activated pathways [27]. ZIKV circumvents this limitation by blocking IFN-β secretion [28], POWV persists in pericytes without completely blocking IFN-β secretion [25] and TBEV and WNV can inhibit the surface expression of the interferon-α/β (IFN-I) receptor (IFNAR1) through prolidase and NS5 interaction [29]. That might explain the persistent infection of HBVPs, despite the upregulation of IFN-β expression, but further data are needed to clarify the susceptibility of HBVP to TBEV infection.

Since TBEV infection in the brain is associated with the induction of cytokines and chemokines, we analyzed paracrine signaling in TBEV-infected HBVPs using Luminex technology. The high expression of various cytokines/chemokines during TBEV infection mediates the immunopathology that can lead to severe disease and increased mortality [30]. In particular, the infected HBVPs predominantly produced IP-10 and RANTES. Both chemokines are critical for the recruitment of activated T cells to sites of tissue inflammation involved in not only viral clearance but also neuropathology in neurotropic flavivirus infections. Elevated levels of these chemokines are found in the CSF of TBE patients, with CD4 T cells showing higher levels of CCR5 compared to blood [31,32,33]. Similar results were reported in TBEV-infected mice [13]. The induction of RANTES by the TBEV NS5 protein leads to the increased infiltration of immune cells into the CNS, contributing to neuroinflammation and brain pathology [34,35]. Moreover, RANTES secretion also promotes the persistent infection of ZIKV-infected HBMEC. ZIKV-elicited CCL5 secretion directs the autocrine HBMEC activation of ERK1/2 survival pathways, supporting ZIKV pathogenesis and spread [36]. A similar signaling pathway may operate in TBEV-infected pericytes, although further experiments are needed to confirm this possibility.

IP-10, which is highly upregulated in the CSF of TBE patients, correlates with the number of T lymphocytes and can activate proinflammatory signaling pathways, affecting CNS cells such as microglia, astrocytes and neurons [37,38].

Our study also found increased CCL2 production by TBEV-infected pericytes, supporting its role in T lymphocyte signaling during TBEV infection. The upregulation of CCL2 in the CSF of TBEV patients and the brains of TBEV-infected mice suggests its importance for immune cell recruitment and viral pathogenesis [13,39,40].

In addition, TBEV-infected HBVPs produced IL-6 and IL-15, which are crucial for regulatory and inflammatory processes in the CNS [41]. Elevated IL-6 levels in the sera of TBE patients and the brains of TBEV-infected mice correlate with increased viral replication and a dysregulated host immune response [42]. IL-6 acts synergistically with IL-7 and IL-15 and increases cytolytic capacity and T-cell proliferation [43].

Differences in host response between the TBEV strains were also observed, particularly in chemokine production. While IP-10 expression remained consistent, RANTES production was significantly higher in Neudoerfl-infected cells, suggesting virus strain-specific influences on the host immune response and disease progression. As these two strains differ in their overall virulence, which could be reflected by the differential immune response of the infected cells, the exact factors determining this differential response remain unknown and will be the subject of our future studies.

This study has several limitations. One key open question, already mentioned above, is what molecular determinants dictate the differential immune response in pericytes infected with TBEV strains differing in virulence. Additionally, the cause of the low susceptibility or permissivity of pericytes to TBEV infection remains unknown. Several factors influence infectivity, including the level of expression of host-cell receptors, the metabolic activity of the cells, the presence or absence of specific enzymes needed for virus replication, and the level of activation of antiviral defense mechanisms. Identifying which of these factors contributes to the low susceptibility or permissivity of pericytes to TBEV infection is an area for future investigation. Furthermore, while we did not observe the formation of a CPE in the TBEV-infected culture, we cannot rule out the possibility of cellular damage or death among the infected cells, which were a minority in the culture. More sensitive methods for detecting such effects will be employed in our future studies. Lastly, it remains unclear whether the soluble factors produced by TBEV-infected pericytes contribute to increased BBB permeability. This will be addressed in future studies involving co-culture experiments with pericytes and other cell components of the NVU.

In summary, this study shows that TBEV can infect the pericytes of the human brain, leading to viral replication and the production of antiviral molecules and chemokines. These findings suggest that pericytes play a role in TBE pathogenesis and may contribute to T-cell recruitment and the inflammatory response in the brain. Further studies, especially in context of interactions with other NVU cells, are needed to elucidate the exact mechanisms and molecular factors by which pericytes influence TBEV infection and disease severity.

## 4. Materials and Methods

### 4.1. Virus and Cells

Two different European TBEV strains were used in this study. The highly virulent strain Hypr was isolated in 1953 in Brno, former Czechoslovakia. The closely related but less virulent strain Neudoerfl was originally isolated from the tick *Ixodes ricinus* in Austria in 1971 and kindly provided by Professor F. X. Heinz, Institute of Virology, Medical University of Vienna, Austria. Prior to this study, both strains were first passaged by the intracranial infection of suckling mice—Hypr eight times; Neudoerfl five times.

Human brain vascular pericytes (HBVP) (ScienCell Cat. No. #1200) were cultured in complete PM medium (ScienCell Cat. No. #1201) at 37 °C in a humidified atmosphere of 5% CO_2_ in air using poly-L-lysine (ScienCell Cat. #0413)-coated culture flasks. For all experiments, HBVPs were used at low passage numbers and always seeded on pre-coated surfaces in complete culture media.

Porcine kidney stable (PS) cells, which were used for the plaque assay, were grown in L-15 medium supplemented with 3% new born calf serum, 1% L-glutamine and 1% Antibiotic–Antimycotic Solution (Biosera, Cholet, France) at 37 °C in ambient air.

### 4.2. Viral Growth

To establish a TBEV growth curve, cells were seeded onto a 96-well plate (5 × 10^4^ cells per well) and infected with the virus at a multiplicity of infection (moi) of 5. After 2 h, cells were washed twice with PBS before fresh culture medium was added. Supernatants were harvested at 1, 3, 6 and 10 days p.i. Additionally, cell monolayers were lysed using an Ambion Cell-to-CT Kit (Invitrogen/Applied Biosystems, Waltham, MA, USA) and further processed for RNA isolation.

### 4.3. Plaque Assay

PS cells were used to determine viral titers, as described previously [44]. Next, 10-fold dilutions of the infectious samples were placed in 96-well plates and incubated with PS cell suspension (5 × 10^4^ cell per well) for 4 h at 37 °C and 0.5% CO_2_. The samples were then covered with a 1:1 (*v*/*v*) overlay mixture (carboxymethylcellulose and a 2× L-15 medium containing 6% PTS, 2% L-glutamine and 2% penicillin/streptomycin/amphotericin B). After 5 days, the plates were washed with PBS and stained with naphthalene black. Virus-produced plaques were counted, and the titers were expressed as PFU/mL.

### 4.4. RNA Isolation

Viral RNA was extracted from TBEV-infected cells at 1, 3, 6 and 10 days p.i.; RNA from non-infected cells served as a control. The RNA was isolated using a QIAamp Viral RNA Mini Kit (Qiagen, Germantown, MD, USA) according to the manufacturer’s instructions. The RNA samples were then stored at −80 °C until the analysis.

Total RNA was extracted from TBEV-infected HBCA and controls using an Ambion Cell-to-CT Kit (Applied Biosystems, Waltham, MA, USA) according to the instructions of the manufacturer. cDNA was synthesized using a High-Capacity RNA-to-cDNA Kit (Applied Biosystems), according to the manufacturer’s protocol. The synthesized cDNAs were used as templates for real-time PCR.

### 4.5. Real-Time qPCR

Following RNA isolation, viral RNA was quantified using a genesig Real-time PCR Detection Kit for TBEV (PrimerDesign Ltd., Eastleigh, UK) using oasig Lyophilized OneStep qRT-PCR (PrimerDesign Ltd., UK) on a Rotor Gene-3000 (Corbett Research, Mortlake, Australia).

Real-time qPCR for host mRNA gene expression was performed as conducted previously [8]. In brief, we used pre-developed TaqMan Reagent Assay IDs, IL1β (Hs01555410_m1), IP10 (Hs01124251_g1), INFβ (Hs01555410_m1) and Oas1 (Hs00973637_m1), with TaqMan Gene Expression Master Mix (Applied Biosystems) on a Rotor Gene-3000 (Corbett Research). Human beta actin (Hs99999903_m1) and glyceraldehyde-3-phosphate dehydrogenase (Hs03929097_g1) housekeeping genes’ RNA transcripts were used for normalization. The amplification conditions were as follows: 2 min at 50 °C (to allow UNG to cleave any contaminating templates); 10 min at 95 °C (to denature UNG and activate the enzymes); 40 cycles of denaturation at 95 °C for 15 s; and annealing/extension at 60 °C for 1 min. To calculate the fold change in gene expression, the CT of the housekeeping gene was subtracted from the CT of the target gene to yield the ΔCT. The change in expression of the normalized target gene was expressed as 2^−ΔΔCT^ where ΔΔCT = ΔCT sample-ΔCT control, as described previously [45].

### 4.6. Bead-Based Multiplex Assay

Concentrations of cytokines, chemokines and growth factors in cell culture supernatant samples were determined using a Human Cytokine/Chemokine Magnetic Bead Panel—Premixed 41 Plex—Immunology Multiplex Assay (HCYTMAG-60K-PX41, Milliplex, EMD Millipore, Burlington, MA, USA), following the manufacturer’s instructions.

### 4.7. Immunocytochemistry

HBVP were seeded in poly-L-lysine-coated wells of glass chamber slides (0.4 cm^2^; 1 × 10^4^ cells per well), infected at a moi of 5 and incubated for 3 days. Non-infected cells served as a control. Cells were then fixed with an ice-cold 1:1 (*v*/*v*) mixture of methanol and acetone for 10 min. After 10 min, cells were washed and blocked with 10% FBS and 5% goat serum for 30 min at 37 °C. Cells were then double-labeled with either rabbit anti-PDGFRβ monoclonal antibody (1:100, MA5-15143, Invitrogen) and mouse anti-flavivirus group antigen monoclonal antibody (1:250, MAB10216, Invitrogen) or rabbit anti-PDIA3 polyclonal antibody (1:250, HPA003230, Sigma-Aldrich, St. Louis, MO, USA) together with anti-flavivirus group antigen antibody. After a 1 h incubation at 37 °C, cells were washed and labeled with goat anti-mouse Alexa Fluor 488 and goat anti-rabbit Alexa Fluor 594 secondary antibodies (1:500, A11029 and A11037, Invitrogen) and incubated for 1 h at 37 °C. After the final wash, coverslips were mounted and counterstained using Fluoroshield with DAPI (Sigma-Aldrich). Images were taken with Olympus BX60 (Tokyo, Japan) and processed using ImageJ 1.53c. All wash steps were performed three times with 0.05% Tween-20 in PBS (PBS-T). Primary antibodies were diluted in the blocking solution, while secondary antibodies were diluted in PBS-T.

### 4.8. Statistical Analysis

Data were analyzed using GraphPad Prism 9.0.2. The data from the multiplex assay were processed as described previously [13]. Net MFI values of the analytes in individual samples instead of concentration were used because of their better fitting to normal distribution. The normality was further improved by log transformation, and the data were analyzed using multiple t-test comparisons with two-stage step-up corrections for multiple comparisons of Benjamini, Krieger and Yekutieli (Q = 2%) to identify statistically significant differences between infected and control samples. All obtained P values were additionally analyzed using a two-stage linear step-up Benjamini, Krieger and Yekutieli procedure (Q = 2%). In the case of concentrations of the analytes tested, the Brown–Forsythe and Welch ANOVA tests with Dunnett’s T3 multiple comparisons test were performed to identify statistically significant differences between different conditions (control, Hypr-infected, Neudoerfl-infected). Differences with *p* < 0.05 were considered significant.

## Data Availability

Data generated in this study are available from the corresponding author upon request.

## Supplementary Materials

The following supporting information can be downloaded via this link: https://www.mdpi.com/article/10.3390/ijms25147892/s1.
