# Supplementary material for: Robust CXCL10/IP-10 and CCL5/RANTES Production Induced by Tick-Borne Encephalitis Virus in Human Brain Pericytes Despite Weak Infection
Source: Int J Mol Sci. 2024 Jul 18;25(14):7892. doi: 10.3390/ijms25147892 (PMC11276942; doi:10.3390/ijms25147892)
Supplement: Supplementary file 1 [file ijms-25-07892-s001.zip › ijms-3074718-supplementary.pdf]

Supplementary Materials

Robust CXCL10/IP-10 and CCL5/RANTES Production Induced by Tick-Borne Encephalitis Virus in Human Brain Pericytes despite Weak Infection

Veronika Prančlová [1,2], Václav Hönig [1,3], Marta Zemanová [1], Daniel Růžek [1,3,4,*] and Martin Palus [1,3,*]

[1] Institute of Parasitology, Biology Centre of the Czech Academy of Sciences, Branisovska 31, CZ-37005 Ceske Budejovice, Czech Republic

[2] Faculty of Science, University of South Bohemia, CZ-37005 Ceske Budejovice, Czech Republic

[3] Laboratory of Emerging Viral Infections, Veterinary Research Institute, Hudcova 70, CZ-62100 Brno, Czech Republic

[4] Department of Experimental Biology, Faculty of Science, Masaryk University, Kamenice 5, CZ-62500 Brno, Czech Republic

* Correspondence: palus@paru.cas.cz

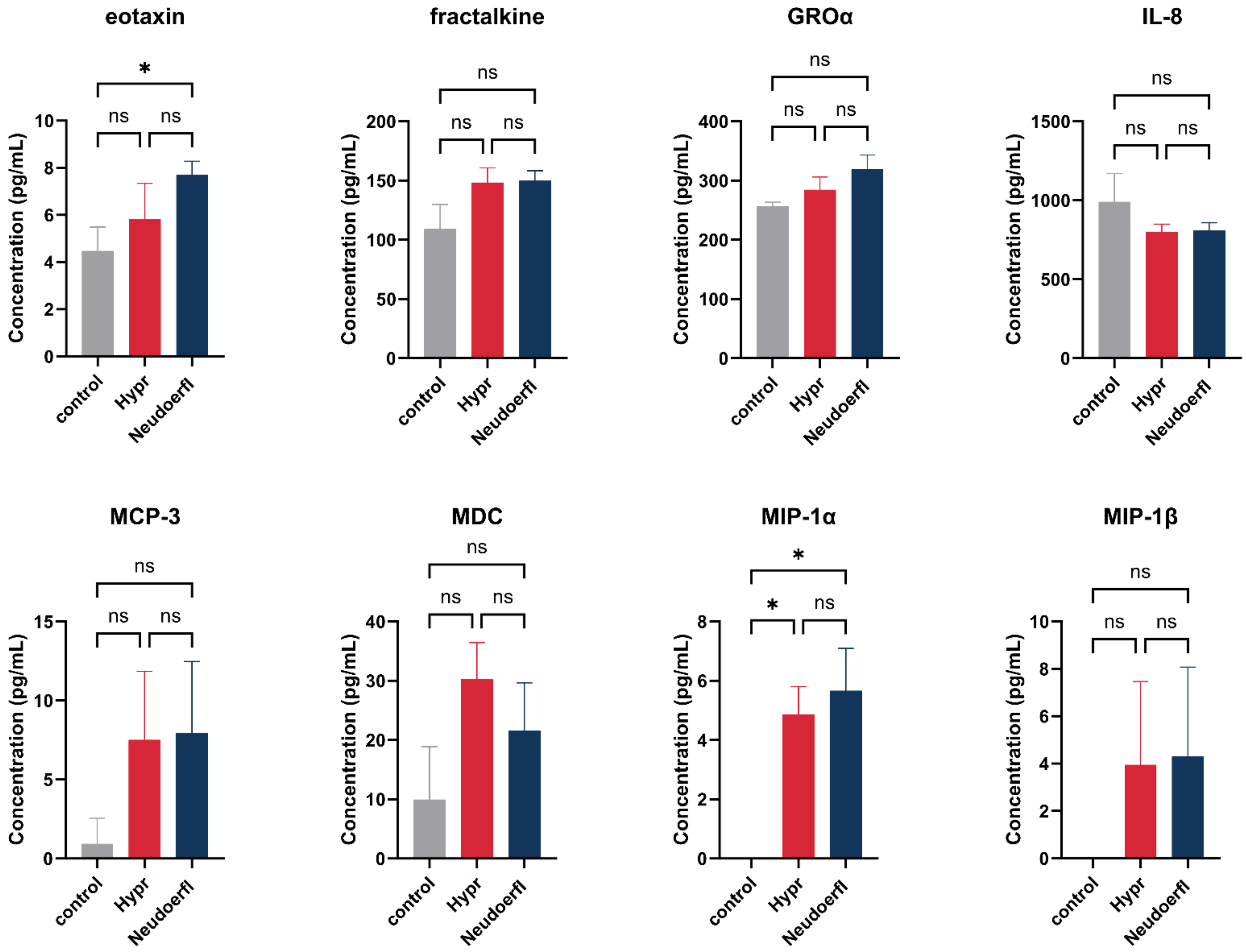

Figure S1. Comparison of TBEV-induced production of chemokines in HBVP infected with different viral strains. Cell-free supernatants of HBVP cells infected with TBEV strains Hypr and Neudoerfl were harvested on day 3 p.i. and used for detection of mmunomodulatory molecules by a micro-bead based multiplex assay The following analytes were monitored: eotaxin (A), fractalkine (B), GROα (C), IL-8 (D), MCP-3 (E), MDC (F), MIP-1α (G), and MIP-1β (H). The results represent mean of biological triplicates +-SD. Statistically significant changes were identified using Brown-Forsythe and Welch ANOVA tests. Statistically significant differences are marked by asterisk (*p<0.05).

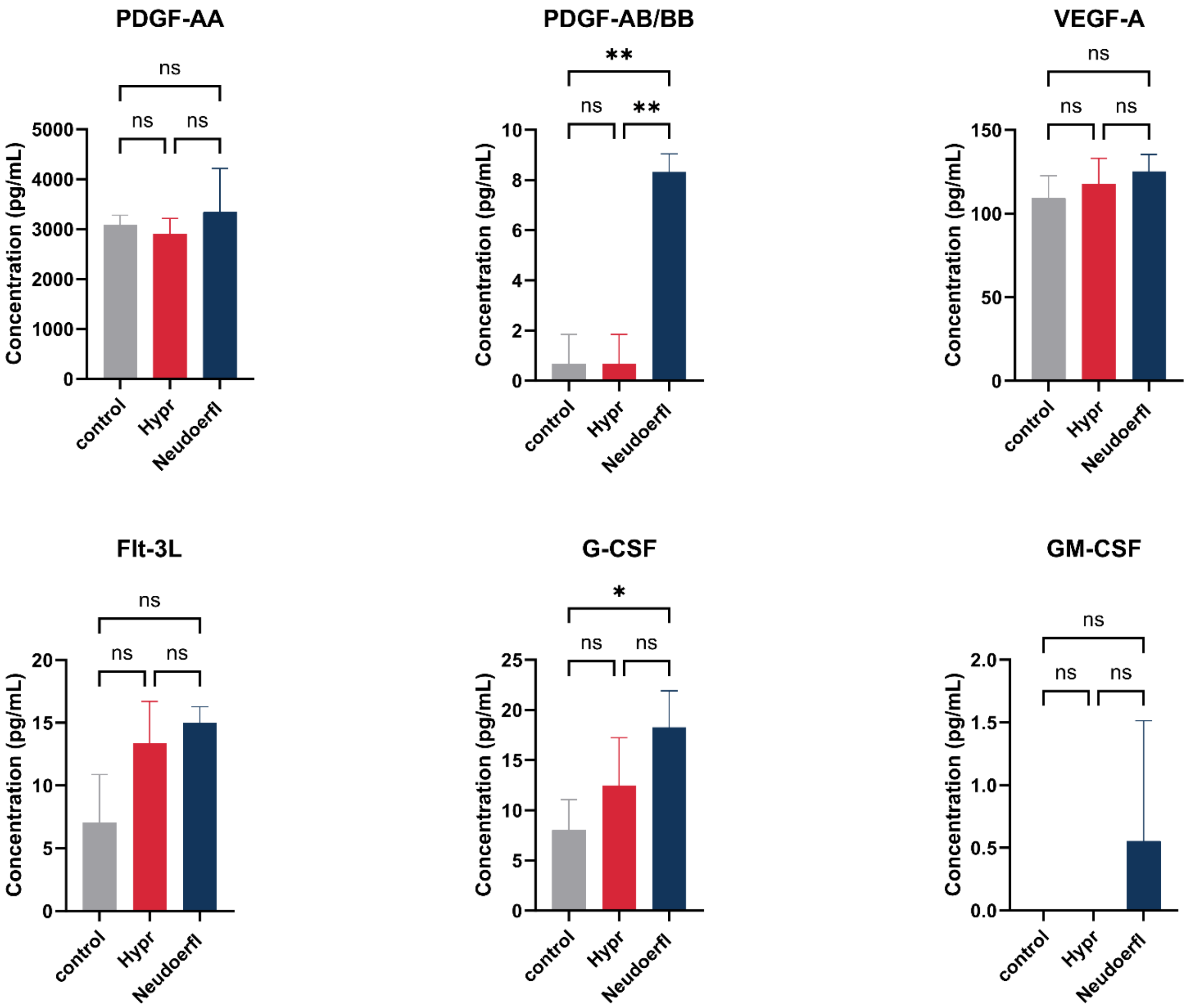

Figure S2. Comparison of TBEV-induced production of colony stimulating factors and growth factors in HBVP infected with different viral strains. Cell-free supernatants of HBVP cultures infected with TBEV strains Hypr and Neudoerfl were harvested on day 3 p.i. and used for detection of immunomodulatory molecules by a micro-bead based multiplex assay. The following analytes were monitored: PDGF-AA (A), PDGF-AB/BB (B), VEGF-A (C), Flt-3L (D), G-CSF (E), and GM-CSF (F). The results represent mean of biological triplicates +-SD. Statistically significant changes were identified using Brown-Forsythe and Welch ANOVA tests. Statistically significant differences are marked by asterisk (*p<0.05, **p<0.01).

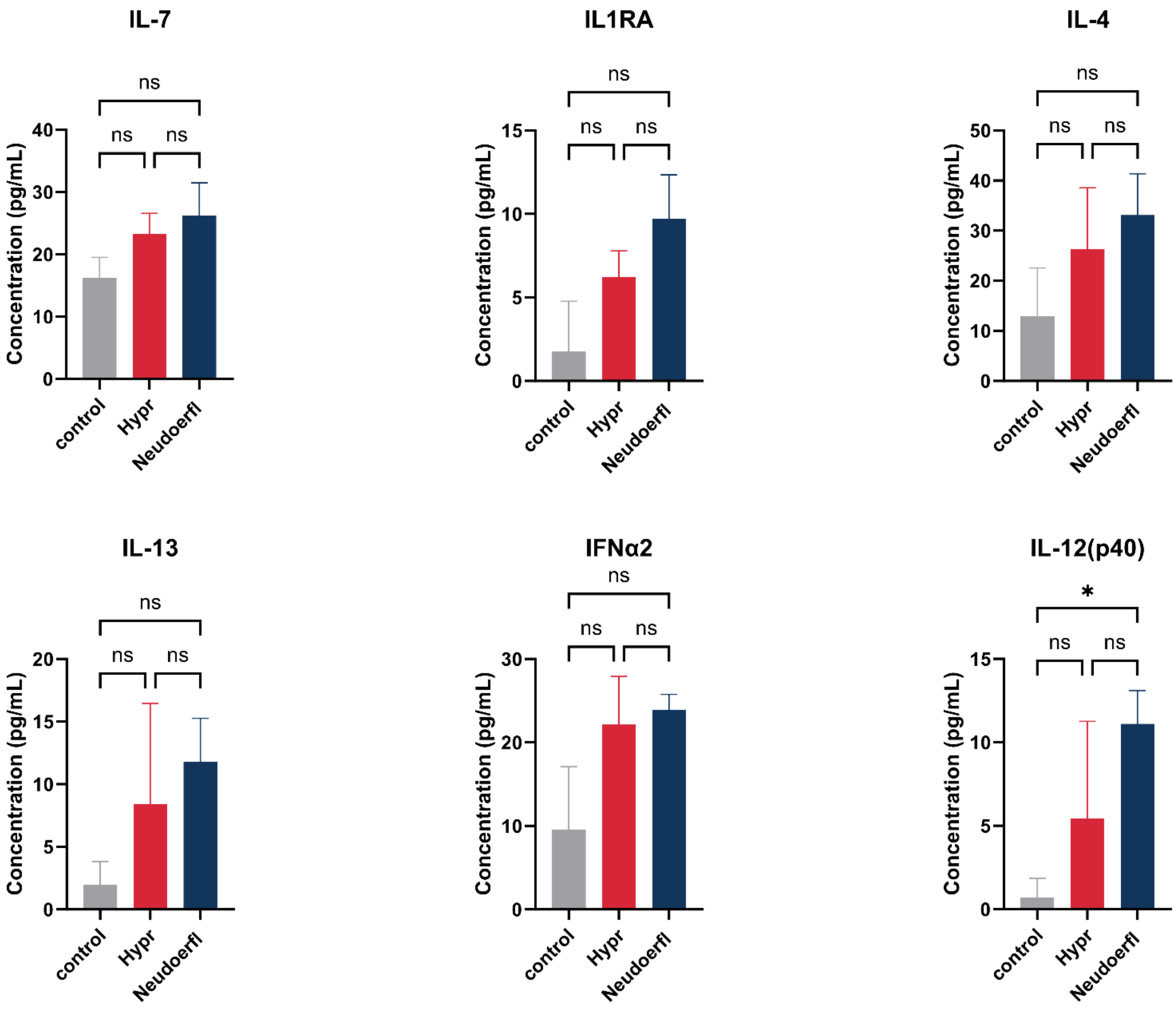

Figure S3. Comparison of TBEV-induced production of cytokines in HBVP infected with different viral strains. Cell-free supernatants of HBVP cultures infected with TBEV strains Hypr and Neudoerfl were harvested on day 3 p.i. and used for detection of modulatory molecules by a micro-bead based multiplex assay. The following analytes were monitored: IL-7 (A), IL1RA (B), IL-4 (C), IL-13 (D), IFNα2 (E), and IL-12(p40) (F). The results represent mean of biological triplicates +-SD. Statistically significant changes were identified using Brown-Forsythe and Welch ANOVA tests. Statistically significant differences are marked by asterisk (*$p<0.05$).
